# The Expression Levels and Clinical Significance of CARDS TX, MyD88, and IL‐8 of Children With Severe *Mycoplasma pneumoniae* Pneumonia

**DOI:** 10.1155/mi/2783518

**Published:** 2026-08-10

**Authors:** Ding Zewen, Hou Jiapu, Sun Ruiyang, Jia Wanyu, Li Peng, Song Chunlan

**Affiliations:** ^1^ Henan Province Engineering Research Center of Diagnosis and Treatment of Pediatric Infection and Critical Care, Children’s Hospital Affiliated to Zhengzhou University, Zhengzhou 450052, Henan, China, zzusah.com

**Keywords:** bronchoalveolar lavage fluid, CARDS TX, IL-8, MyD88, severe *Mycoplasma pneumoniae* pneumonia

## Abstract

**Objective:**

To investigate the expression levels of community‐acquired respiratory distress syndrome toxin (CARDS TX), myeloid differentiation factor 88 (MyD88), and interleukin‐8 (IL‐8) in the bronchoalveolar lavage fluid (BALF) of children with severe *Mycoplasma pneumoniae* pneumonia (SMPP) and explore their clinical significance in the pathogenesis of SMPP.

**Methods:**

This was a retrospective analysis of clinical data from 29 patients with SMPP and 12 patients with general *Mycoplasma pneumoniae* pneumonia (GMPP). Moreover, the expression levels of CARDS TX, MyD88, and IL‐8 in the BALF of the two groups were detected and compared.

**Results:**

The expression levels of CARDS TX, MyD88, and IL‐8 were positively correlated with SMPP (*p*  < 0.05). Univariate logistic regression analysis indicated that CARDS TX, MyD88, and IL‐8 are potential risk factors for SMPP (*p*  < 0.05). The areas under the curve for the CARDS TX, MyD88, and IL‐8 assays were 0.857, 0.730, and 0.799, respectively. The optimal cutoff values were 10.91 ng/mL, 2.21 ng/mL, and 120.52 pg/mL, respectively.

**Conclusions:**

Elevated levels of CARDS TX, MyD88, and IL‐8 in BALF all demonstrate diagnostic utility for children with SMPP, with CARDS TX exhibiting the highest diagnostic sensitivity. A CARDS TX concentration >10.91 ng/mL, a MyD88 concentration >2.21 ng/mL, and an IL‐8 concentration >120.52 pg/mL are potential risk factors for SMPP.

## 1. Introduction

*Mycoplasma pneumoniae* (MP) is a common pathogen that causes respiratory infections worldwide. Studies indicate that the incidence of MP infections exhibits cyclical patterns in multiple countries, with epidemics recurring every 3–5 years [1]. *Mycoplasma pneumoniae* pneumonia (MPP) primarily affects children over 5 years of age and accounts for 20%–40% of community‐acquired pneumonia (CAP) cases [2]. MPP is predominantly a self‐limiting infection with a favorable prognosis. However, if not diagnosed early and treated appropriately, it may progress to severe pneumonia with potentially fatal consequences [3]. In recent years, the incidence of severe MPP (SMPP) has increased [4], which poses a serious threat to children’s health. Patients with SMPP may develop complications such as pleural effusion and atelectasis and may even rapidly progress to respiratory failure or life‐threatening extrapulmonary complications, resulting in organ and system involvement in children and the development of long‐term sequelae [5]. SMPP not only presents with severe symptoms and a prolonged course but is also associated with multiple complications and sequelae, such as atelectasis, obstructive bronchiolitis, and other related conditions. It has been reported that between 2014 and 2020, 16.0% of pediatric patients with MPP in Suzhou, China, progressed to severe cases. In North China, the annual incidence of SMPP increased significantly from 0.7% in 2007 to 42.6% in 2016 [6].

The severity of MPP is related to the virulence factors of MP and the host immune response. It has been reported that community‐acquired respiratory distress syndrome toxin (CARDS TX) is synthesized during MP infection and is closely associated with the replication and persistence of MP, airway inflammation, and pathological changes in lung tissue [7]. The CARDS TX produced by MP can induce a robust lymphocyte response [8]. Myeloid differentiation factor 88 (MyD88) is a signal hub for the inflammatory response following MP infection and plays a crucial role in the process by which macrophages clear MPs from the lungs [9]. The CARDS TX can promote the secretion of interleukin‐8 (IL‐8), and MP can also act directly on macrophages to induce the release of IL‐8 [10].

Bronchoalveolar lavage fluid (BALF), in essence, is an eluate from the surface of diseased airways and alveoli and can directly reflect the inflammatory and immune responses in the local lung tissue affected by infection. Previous studies mainly focused on clinical baseline data and routine inflammatory indicators, and a single biomarker shows limited predictive efficacy. In this study, we combined CARDS TX, MyD88, and IL‐8 in BALF to establish a prediction model and comprehensively evaluated the risk of SMPP onset and progression from the perspectives of virulence factor, immune pathway protein, and inflammatory chemokine. It is helpful for pediatricians to achieve early identification of SMPP and, at the same time, provide new insights for the diagnosis and treatment of SMPP.

## 2. Materials and Methods

### 2.1. Research Subjects

We retrospectively recruited children diagnosed with MPP hospitalized in our hospital from June to December 2023, who underwent bronchoalveolar lavage (BAL) with BALF specimens collected. All subjects were divided into the SMPP group and the general MPP (GMPP) group in accordance with the diagnostic criteria specified in the *Guidelines for the Diagnosis and Treatment of Mycoplasma pneumoniae Pneumonia in Children* (*2023 Edition*) [11].

### 2.2. Inclusion Criteria

(1) Patients aged less than 18 years old with confirmed MPP. (2) Patients who underwent BAL. (3) Specimen collection was conducted with parental informed consent.

### 2.3. Diagnostic Criteria for MPP (11)

A confirmed diagnosis can be established if all the following conditions are satisfied: (1) Presence of clinical manifestations including fever, cough, tachypnea, and abnormal lung breath sounds. (2) Typical chest imaging manifestations such as interstitial infiltration, segmental or lobar consolidation, and hilar lymphadenopathy. (3) Positive results of the MP nucleic acid or antibody test.

### 2.4. Diagnostic Criteria for SMPP (11)

Meeting any one of the following criteria constitutes severe disease: (1) persistent high fever (≥39°C) for ≥5 days or fever for ≥7 days, with no decrease or even an increase in peak body temperature, resulting in high fever. (2) Wheezing, shortness of breath, difficulty breathing, chest pain, or hemoptysis were present. (3) Although extrapulmonary complications have developed, they do not meet the criteria for critical illness. (4) At rest, the pulse oxygen saturation level is ≤0.93 when breathing air at sea level. (5) There was a significant increase in CRP, LDH, or D‐dimer. (6) High‐density pulmonary consolidation involving more than two‐thirds of a lung lobe or high‐density consolidation in two or more lobes (regardless of the extent of involvement), with unilateral or bilateral diffuse bronchiolitis, may be associated with bronchitis. (7) The clinical symptoms progressively worsen, and imaging findings show that the lesion range progresses by more than 50% within 24–48 h.

### 2.5. Exclusion Criteria

(1) Individuals with congenital or chronic cardiopulmonary disease, immunodeficiency, or a history of recurrent respiratory infections. (2) Patients with incomplete medical records.

### 2.6. Data Collection

The following data of the children were collected: age, gender, duration of fever, length of hospital stay, bronchoscopic findings and CT manifestations, white blood cell count, neutrophil‐to‐lymphocyte ratio, C‐reactive protein, lactate dehydrogenase, procalcitonin, IL‐6, and erythrocyte sedimentation rate.

### 2.7. The Use of Antibiotics and Systemic Glucocorticoid

All enrolled children received standardized anti‐infective and anti‐inflammatory treatment per the Guidelines for the Management of Community‐Acquired Pneumonia in Children. Initial empirical therapy consisted of macrolides or cephalosporins for indicated patients; an antibiotic step‐up regimen was used for a poor clinical response. Intravenous methylprednisolone sodium succinate (1–2 mg/[kg·d]) was given for systemic anti‐inflammation, with individualized treatment duration based on clinical symptoms, inflammatory markers, and imaging results. Expectorants, bronchodilators, rehydration, and other supportive agents were administered as needed.

### 2.8. BALF Collection

After signing the informed consent form, the procedure was performed in accordance with the *Guidelines for Flexible Bronchoscopy in Chinese Pediatrics* (*2018 Edition*) [12]. Bronchoscopy was performed, and the patient’s BALF was collected. After centrifugation, the samples were stored at −80°C for future use [13].

### 2.9. Detection of Indicators in the BALF

CARDS TX, MyD88, and IL‐8 expression levels in the BALF were detected via an ELISA according to the manufacturer’s instructions. (CARDS TX ELISA Kit, Shanghai Enzyme‐Linked Biotechnology Co., Ltd.), (MyD88 Rapid Detection ELISA Kit, Wuhan Fine Biotech Co., Ltd.), (IL‐8 ELISA Kit, Elabscience Biotechnology Co., Ltd.).

### 2.10. Statistical Methods

Analysis was performed via the SPSS 27.0 statistical software package. The normality test was performed via the Kolmogorov‒Smirnov test. Normally distributed data are expressed as the mean ± standard deviation (*X̅* ± *S*); nonnormally distributed data are expressed as the median (interquartile range) (M [P25, P75]). For intergroup comparisons of measurement data, the independent sample *t*‐test was used for normally distributed data, whereas the Mann–Whitney *U* test was used for nonnormally distributed data. Enumeration data are expressed as percentages or rates, and the chi‐square test (*χ* ^2^) was used for intergroup comparisons. Pearson correlation analysis was applied to normally distributed variables, and Spearman rank correlation analysis was utilized for nonnormally distributed variables. Univariate logistic regression analysis was used to identify potential risk factors for SMPP. The area under the receiver operating characteristic (ROC) curve (AUC) was used to evaluate the predictive value of the indicators. A *p* value <0.05 was considered statistically significant.

## 3. Results

### 3.1. General Information

**Figure 1:**
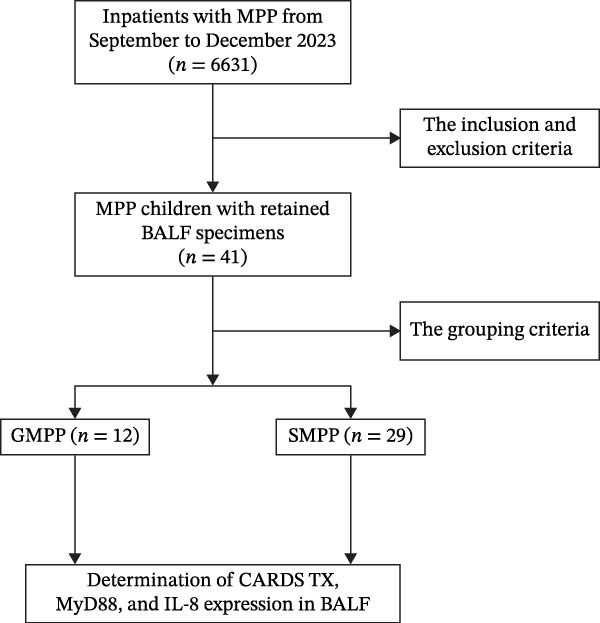
Flowchart of participant enrollment.

This study included a total of 41 pediatric patients with MPP, comprising 29 patients in the SMPP group and 12 patients in the GMPP group. The flowchart is shown in Figure 1. There were no significant differences between the two groups of pediatric patients in terms of the ratio and age (*p*  > 0.05). The days of hospitalization were 10.00 (9.00, 12.00) days in the SMPP group and 7.00 (7.00, 10.50) days in the GMPP group, with significant intergroup difference (*p*  < 0.05). The mean duration of fever onset in the SMPP group was 7.24 ± 3.77 days, whereas that in the GMPP group was 4.25 ± 3.19 days, indicating a statistically significant difference (*p*  < 0.05). There was no significant difference in the incidence of mixed respiratory pathogen infection between the two groups (*p* > 0.05) (Table 1).

**Table 1:** Comparison of general information and laboratory tests.

Variables		GMPP (*N* = 12)	SMPP (*N* = 29)	*Z*	*p*
Male^*χ*^ (*N*, %)		7 (58.33%)	13 (44.83%)	0.620	0.431
Days of hospitalization^U^ (days)		7 (7.00, 10.50)	10.00 (9.00, 12.00)	−2.215	0.027
Duration of fever^t^ (days)		4.25 ± 3.19	7.24 ± 3.77	−2.411	0.021
Age (years)		7.45 ± 2.01	8.06 ± 2.93	−0.631	0.528
CARDS TX^t^ (ng/mL)		8.14 ± 3.95	12.84 ± 2.92	−3.811	<0.001
MYD88^U^ (ng/mL)		1.65 (0.65, 2.20)	2.21 (1.60, 4.05)	−2.226	0.026
IL8^U^ (pg/mL)		85.72 (69.67, 171.99)	202.94 (147.54, 377.31)	−2.893	0.004
WBC^U^ (10^9^/L)		9.27 (7.91, 10.63)	8.15 (5.89, 11.36)	−0.802	0.422
Neu^U^ (%)		57.8 (52.15, 75.16)	69.4 (62.70, 75.55)	−1.318	0.187
NLR^U^		1.93 (1.36, 4.46)	3.14 (2.31, 4.11)	−1.117	0.264
CRP^U^ (mg/L)		14.10 (7.28, 22.80)	12.17 (5.71, 27.82)	−0.122	0.903
PCT^U^ (ng/mL)		0.08 (0.05, 0.16)	0.08 (0.04, 0.11)	−0.292	0.770
IL‐6^U^ (pg/mL)		8.41 (3.49, 23.67)	12.24 (5.30, 32.04)	−1.199	0.230
ESR^U^ (mm/h)		38.50 (9.75, 58.75)	38.00 (29.25, 65.00)	−0.815	0.415
LDH^U^ (U/L)		242.50 (219.00, 294.25)	291.00 (251.00, 401.00)	−1.704	0.088
Mixed infection^*χ*^		3 (25.00%)	10 (34.48%)	0.352	0.553
Phlegm plug obstruction^*χ*^ (*N*, %)		0	7 (24.13%)	1.996	0.158
Plastic bronchitis^*χ*^ (*N*, %)		0	7 (24.13%)	1.996	0.158
Pulmonary consolidation^*χ*^ (*N*, %)	None	1 (8.33%)	2 (6.90%)	−1.186	0.236
	1 Lung lobe	10 (83.33%)	19 (65.52%)		
	Multiple lung lobes	1 (8.33%)	8 (27.59%)		
Bronchial stenosis^*χ*^ (*N*, %)		1 (8.33%)	5 (17.24%)	0.062	0.804
Pleural effusion^*χ*^ (*N*, %)		2 (16.67%)	8 (27.59%)	0.116	0.733
Atelectasis^*χ*^ (*N*, %)		0	5 (17.24%)	1.021	0.312
Pleural thickening^*χ*^ (*N*, %)		0	7 (24.13%)	1.996	0.158
Macrolides combined with cephalosporins^*χ*^ (*N*, %)		9 (75.00%)	23 (79.30%)	0.092	0.762
Antibiotic escalation therapy^*χ*^ (*N*, %)		5 (41.65%)	15 (51.72%)	0.344	0.558
Methylprednisolone sodium succinate^U^ (days)		4.00 (3.25, 4.75)	4.00 (3.00, 6.00)	−0.296	0.767

*Note: t*: independent samples *t*‐test; U: Mann–Whitney U test; χ: chi‐square test.

### 3.2. The Use of Antibiotics and Systemic Glucocorticoid

Prior to BAL, 9 patients in the GMPP group and 23 in the SMPP group received combined macrolide and cephalosporin therapy, while an antibiotic step‐up regimen was implemented in 5 and 15 patients, respectively. No statistically significant between‐group differences were detected for both parameters (*p*  > 0.05). Children in both groups received systemic anti‐inflammatory corticosteroid therapy, and the duration of methylprednisolone sodium succinate treatment was comparable between the two groups (*p*  > 0.05) (Table 1).

### 3.3. Comparison of Bronchoscopic Findings and CT Findings

There was no significant difference between the two groups of pediatric patients in terms of the proportion of sputum plug obstruction and plastic bronchitis observed during bronchoscopy (*p*  > 0.05). There was no significant difference in the proportion of CT findings showing pulmonary consolidation, bronchial stenosis, pleural effusion, atelectasis, or pleural thickening (*p*  > 0.05). (Table 1).

### 3.4. Comparison of Laboratory Test Results

There were no significant differences in the white blood cell count, neutrophil percentage, neutrophil‐to‐lymphocyte ratio, C‐reactive protein, procalcitonin, IL‐6, erythrocyte sedimentation rate, and lactate dehydrogenase between the two groups (*p*  > 0.05). The concentrations of CARDS TX, MyD88, and IL‐8 in the BALF of the SMPP group were 12.84 ± 2.92 ng/mL, 2.21 (1.60, 4.05) ng/mL, and 202.94 (147.54, 377.31) pg/mL, respectively. The expression levels of these three indicators were significantly greater than those in the GMPP group (*p*  < 0.05) (Table 1).

### 3.5. Correlation Analysis

Correlation analysis was performed between CARDS TX, MyD88, and IL‐8 expression levels and SMPP. The results revealed that CARDS TX, MyD88, and IL‐8 expression levels were positively correlated with SMPP (*p*  < 0.05). (Table 2).

**Table 2:** Correlation analysis with SMPP.

Variables	*r*	*p*
CARDS TX^P^ (ng/mL)	0.565	<0.001
MYD88^S^ (ng/mL)	0.356	0.024
IL‐8^S^ (pg/mL)	0.463	0.003

*Note:* P: Pearson correlation analysis; S: Spearman rank correlation analysis.

### 3.6. Univariate Logistic Regression Analysis

Univariate logistic regression analysis revealed that CARDS TX, MyD88, and IL‐8 are potential risk factors for SMPP (*p*  < 0.05), with odds ratios (ORs) of 1.544, 2.298, and 1.015, respectively. (Table 3).

**Table 3:** Univariate logistic analysis.

Variables	Univariate logistic regression		
	OR	95% CI	*p*
CARDS TX (ng/mL)	1.544	(1.106, 2.155)	0.011
MYD88 (ng/mL)	2.298	(1.049, 5.034)	0.038
IL8 (pg/mL)	1.015	(1.003, 1.027)	0.018

### 3.7. ROC Curve

**Figure 2:**
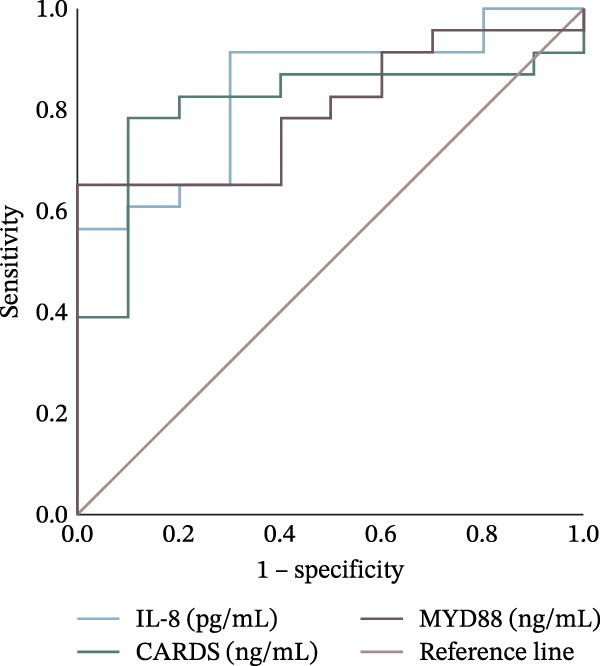
ROC curves of predictive indicators.

ROC curve analysis was performed for the CARDS TX, MyD88, and IL‐8 expression levels. The results indicated that CARDS TX demonstrated the highest predictive performance (AUC = 0.857), followed by IL‐8 (AUC = 0.799) and MyD88 (AUC = 0.730). The optimal cutoff values for the three indicators mentioned above are 10.91 ng/mL, 120.52 pg/mL, and 2.21 ng/mL (Figure 2 and Table 4).

**Table 4:** Results of the ROC curve.

Variables	AUC	Cut‐off value	Youden index	Sensitivity	Specificity	95% CI	*p*
CARDS TX(ng/mL)	0.857	>10.91	0.6826	78.26	90.00	(0.716, 0.997)	0.001
MYD88(ng/mL)	0.730	>2.21	0.5172	51.72	100.00	(0.571, 0.890)	0.026
IL8(pg/mL)	0.799	>120.52	0.5549	82.76	72.73	(0.656, 0.943)	0.004

## 4. Discussion

In our study, there were no differences in the gender ratio or age between the SMPP group and the GMPP group, and the mean age of the children in both groups was within the school‐age range. Previous studies have indicated that severe Mycoplasma pneumonia occurs commonly in children aged 2–4 years as well as those over 5 years old [8], which is consistent with our results. Our study also found that children in the SMPP group had longer fever duration and longer hospital stay, this is consistent with the findings of previous studies [14], this also indicates severe inflammatory infiltration and damage to the lung tissue of the SMPP group, accompanied by an excessive systemic inflammatory response. Therefore, clinical management requires not only antimicrobial therapy but also combined anti‐inflammatory treatment. The SMPP group showed significantly elevated levels of CARDS TX, MyD88, and IL‐8 in BALF. CARDS TX >10.91 ng/mL, MyD88 >2.21 ng/mL, and IL‐8 > 120.52 pg/mL in BALF are potential risk factors for SMPP, and each demonstrates diagnostic utility for this condition. Elevated BALF CARDS TX, MyD88, and IL‐8 above the above cut‐off values indicate a markedly increased risk of progression to severe Mycoplasma pneumonia in children. These markers can be used as early warning indicators to facilitate early disease assessment, timely anti‐inflammatory intervention, optimize clinical strategies, and reduce the occurrence of severe complications.

CARDS TX is a specific virulence factor associated with the pathogenesis of MP, and its expression level is positively correlated with the severity of pulmonary disease [15]. MPs can directly release CARDS TX to damage host cells [16]. The main mechanism is that the ADP‐ribosyltransferase activity of CARDS TX interferes with the regulation of intracellular signal transduction, the release of inflammatory factors, and the disruption of cellular homeostasis, ultimately leading to apoptosis. In addition, CARDS TX can induce irreversible vacuolization of the host cell cytoplasm, causing cellular metabolism, immune dysfunction, and organelle dysfunction [7]. Research indicates that following MP infection, CARDS TX can be detected in respiratory epithelial cells and the bronchioloalveolar space [17]. We hypothesize that since MP primarily colonizes the mucosa of the respiratory tract—specifically the bronchi, bronchioles, and alveolar ducts—the airway epithelial cells and alveolar epithelial cells in the SMPP group presented more severe damage.

Previous studies have demonstrated that elevated levels of CARDS TX in BALF serve as a predictive factor for refractory mycoplasma infection [16]. Similarly, our study suggests that elevated levels of CARDS TX in the BALF represent a potential risk factor for SMPP. Elevated CARDS TX levels in BALF indicate more severe lung damage caused by MP through CARDS TX virulence, suggesting that MPP may be more likely to progress to SMPP. Reports indicate that CARDS TX expression is elevated within host cells during the early stages of MP infection. Experimental analysis of BALF from MP‐infected mice revealed that CARDS TX levels peaked 1 day postinfection and subsequently declined gradually but remained detectable at 35 days postinfection [17]. These findings suggest that we need to dynamically monitor CARDS TX levels in BALF to better understand the disease progression caused by MP. Notably, CARDS TX is immunogenic, making it a potential candidate for vaccine antigens [18]. Research has shown that when rCARDS TX is used as a vaccine antigen, BALF from mice immunized with rCARDS TX showed significantly reduced numbers of macrophages and inflammatory cytokines [18]. Future development of vaccines targeting CARDS TX may effectively delay the progression of MPP to SMPP.

Research has suggested that MyD88 activates NFκB and multiple interferon regulatory factors by transducing Toll‐like receptor (TLR) signals and that MyD88‐NFκB signaling plays a crucial role in the clearance of MP by macrophages [19]. After infection, macrophages are recruited and activated locally to phagocytose and clear MPs, suggesting that the MyD88 signaling pathway plays an important role in this process. Additionally, MyD88 can interact with TLR2 and TLR4 to activate downstream pathways, leading to the massive release of proinflammatory cytokines [20, 21]. MyD88 serves as a signaling hub in inflammatory responses and may also represent a therapeutic target for numerous inflammatory diseases. Research has already demonstrated that inhibiting MyD88 can effectively treat inflammatory lung diseases [22]. For example, bicyclol reduces the levels of inflammatory cytokines and decreases the number of inflammatory cells in lung tissue by inhibiting MyD88, thereby alleviating the symptoms of acute lung injury [23]. Previous studies have shown that the relative expression levels of TLR2 and MyD88 in the BALF of children in the MPP group were significantly greater than those in the control group [9]. Similarly, in our study, the MyD88 expression levels in the BALF were significantly greater than those in the control group, and the expression level of MyD88 was positively correlated with SMPP, suggesting that the progression of MPP to SMPPMyD88 may participate in the inflammatory pathophysiology of SMPP by influencing macrophage activation and proinflammatory cytokine release and that its expression levels may reflect disease severity.

Previous studies have reported elevated serum IL‐8 concentrations in patients with SMPP [24]. In our study, IL‐8 expression levels in the BALF of SMPP patients were also significantly elevated. These findings suggest that IL‐8, an inflammatory mediator, is involved in the inflammatory response mechanism of MP and plays a crucial role in the progression of MP to SMPP; SMPP patients exhibit intense inflammatory responses and more severe diseases. IL‐8 is a chemokine that induces neutrophil infiltration, recruiting neutrophils to migrate to pulmonary lesions [25]. We believe that this process forms a vicious cycle of “inflammatory infiltration ‐ tissue damage ‐ exacerbated inflammation,” which is an important cause of complications such as pulmonary consolidation and pleural effusion in SMPP. LDH is released extracellularly when cell lysis or cell membrane damage occurs, leading to increased LDH levels [26].As an indicator for monitoring cellular and tissue injury, serum LDH levels are markedly elevated in children with SMPP [27]. Theoretically, IL‐8 and LDH should rise in parallel during inflammatory progression. However, our results revealed a significant intergroup difference only in IL‐8, while LDH showed no statistical difference. We acknowledge that the relatively small sample size is the primary cause of this inconsistency. Additionally, all enrolled children were inpatients receiving BAL, who generally presented with relatively severe illness. As such, baseline LDH values were naturally elevated in the GMPP group, further diminishing the between‐group difference. Although the between‐group difference in LDH did not reach statistical significance (*p* = 0.088), the *p* value was close to the critical threshold for significance. Moreover, the median LDH level was higher in the SMPP group than in the GMPP group, which still bears certain clinical implications, supporting that LDH levels are inclined to increase in children diagnosed with SMPP.

Research has shown that CARDS TX can activate various downstream signaling pathways through the MyD88 pathway, leading to inflammatory and immune responses [9]. Therefore, we propose that CARDS TX may increase IL‐8 release via the MyD88 pathway. Following MP infection, not only does the expression of cytokines such as IL‐8 increase within the respiratory tract but neutrophils and macrophages are also rapidly recruited to lung tissue and the airways [19]. Therefore, CARDS TX, various immune cells, and cytokines such as IL‐8 all participate in the pathological process of SMPP. Notably, as MPP disease progresses, the proportion of neutrophils in the lungs increases significantly [28], and excessive activation of neutrophils leads to tissue damage [29]. Necrotic tissue itself can act as an inflammatory signal to activate immune cells, triggering a vicious cycle of “damage ‐ inflammation ‐ further damage.” In the future, we will further investigate the effect of CARDS TX on the release of cytokines such as IL‐8 in children with SMPP as well as the relationship between cytokines and the number of immune cells.

## 5. Limitations

This study has certain limitations. First, as a retrospective study, it cannot completely exclude confounding factors. We only adopted qualitative MP diagnosis and failed to measure BALF MP load, so intergroup differences in CARDS TX and other indicators could not be analyzed after adjusting for the MP load. Second, the sample size of this study was relatively small, and the sample source is single, resulting in a lack of broad representativeness. Additionally, bronchoalveolar lavage is a relatively special diagnostic and therapeutic procedure, and the number of patients meeting the inclusion criteria in clinical practice is limited—these factors make it difficult to generalize the conclusions to clinical practice. Therefore, it is necessary to expand the sample size and conduct multicenter collaborative studies with quantitative MP load detection for further verification.

## 6. Conclusions

In conclusion, the pathological mechanism of SMPP in children involves the synergistic effect of three factors: the toxic damage caused by CARDS TX, signal transduction with MyD88 as the signal hub, and the excessive local inflammatory response mediated by cytokines such as IL‐8. The high expression of CARDS, TX, MyD88, and IL‐8 in BALF not only represents a key characteristic of SMPP but also serves as an auxiliary diagnostic marker, disease assessment indicator, and potential therapeutic target for SMPP.

## Funding

This work was supported by the Henan Provincial Science and Technology Research Project (Grant 252102311119).

## Ethics Statement

The studies involving humans were approved by the Medical Ethics Committee of Henan Children’s Hospital (2025‐KY‐0037‐001). The studies were conducted in accordance with the local legislation and institutional requirements. Written informed consent for participation in this study was provided by the participants’ legal guardians/next of kin.

## Conflicts of Interest

The authors declare no conflicts of Interest.

## Data Availability

The data that support the findings of this study are available from the corresponding author upon reasonable request.
